# Dr. Leroy Wendell King

**Published:** 1912-07

**Authors:** 


### OBITUARY

Dr. Leroy Wendell King of Lowville, N. Y., died at the Ogdensburg State Hospital on May 29, aged 37, four weeks after an appendix operation. He was graduated at the University of Michigan, 1899, and was President of the Lewis Co. Medical Society.

